# Dopamine/2-Phenylethylamine Sensitivity of Ion-Selective Electrodes Based on Bifunctional-Symmetrical Boron Receptors

**DOI:** 10.3390/s19020283

**Published:** 2019-01-12

**Authors:** Martyna Durka, Krzysztof Durka, Agnieszka Adamczyk-Woźniak, Wojciech Wróblewski

**Affiliations:** 1 Chair of Medical Biotechnology, Faculty of Chemistry, Warsaw University of Technology, Noakowskiego 3, 00-664 Warsaw, Poland; mjanczyk@ch.pw.edu.pl; 2 Department of Physical Chemistry, Faculty of Chemistry, Warsaw University of Technology, Noakowskiego 3, 00-664 Warsaw, Poland; kdurka@gmail.com (K.D.); agnieszka@ch.pw.edu.pl (A.A.-W.)

**Keywords:** ion-selective electrodes, organoboron receptors, dopamine

## Abstract

Piperazine-based compounds bearing two phenylboronic acid or two benzoxaborole groups (PBPA and PBBB) were applied as dopamine receptors in polymeric membranes (PVC/DOS) of ion-selective electrodes. The potentiometric sensitivity and selectivity of the sensors towards dopamine were evaluated and compared with the results obtained for 2-phenylethylamine. Since the developed electrodes displayed strong interference from 2-phenylethylamine, single-molecule geometry optimizations were performed using the density functional theory (DFT) method in order to investigate the origin of dopamine/2-phenylethylamine selectivity. The results indicated that phenylboronic acid and benzoxaborole receptors bind dopamine mainly through the dative B–N bond (like 2-phenylethylamine) and the potentiometric selectivity is mainly governed by the higher lipophilicity of 2-phenylethylamine.

## 1. Introduction

Nowadays, boronic acids are important group of compounds widely used in synthetic and medicinal chemistry, i.e., Suzuki-Miyaura cross-coupling reactions [1], protection of diols [2] and selective reduction of aldehydes [3]. Due to their specific properties, boronic acids have also been proposed for the development of enzyme inhibitors [4], boron neutron capture therapy (BNCT) agents [5] as well as feedback-controlled drug delivery polymers [6]. Since boronic acids interact with 1,2- or 1,3-diol-containing species to rapidly and reversibly form five- or six-membered rings, the identification and determination of diols has been a subject of interest over the past few decades. However, the majority of the developed methods involve spectroscopic detection. Specifically, such receptors have been applied for the selective recognition of molecules containing diol functionality, including saccharides [7,8,9,10,11] and selected neurotransmitters [12,13].

Recently, much attention has been paid to benzoxaboroles—a group of internal hemiesters of boronic acids consisting of a conjugated phenyl ring and a five-membered oxaborole ring [14,15,16,17]. Apart from their wide application in medicine [18,19,20,21], they have attracted much attention due to their excellent diol-binding abilities [22,23,24,25]. The incorporation of the boron atom into the cyclic system induced strains around the boron coordination sphere, thus increasing boron Lewis acidity and causing *p*K_a_ depression. Consequently, benzoxaboroles are able to bind diols under neutral physiological conditions. Another important class of diol receptors are ortho-substituted aminomethyl arylboronic acids (so-called Wulff-type receptors) [26,27]. Under neutral aqueous conditions, such species tend to form intramolecular B–N interactions, leading to the rehybridization of the boron center from trigonal to tetrahedral geometry, and thus significantly increasing their affinity towards diols. Further extensive synthetic works on benzoxaborole and aminomethyl boronic acids resulted in the improvement of their diol affinity and selectivity towards specific groups of analytes. One of these strategies relied on the involvement of two boronic functionalities in the molecule [28,29,30,31,32], and special emphasis has been placed on piperazine based bis-boronic acids [33,34]. For instance, Anslyn et al. used such receptors as components of indicator displacement sensor arrays for the discrimination and classification of ginsenosides and ginseng extracts [35,36].

Dopamine (DA) is an important neurotransmitter in mammalian central nervous systems and its deficit results in brain disorder, such as Parkinson’s disease, whereas its functional excess or the oversensitivity of certain DA receptors has been implicated as the cause of schizophrenia [37]. The routine analysis of catecholamine neurotransmitters in body fluids is commonly performed using high-performance liquid chromatography or capillary electrophoresis [38]. On the other hand, the determination of neurotransmitters such as dopamine by direct electrochemical techniques is quite challenging. Consequently, various attempts based on polymer-modified electrodes, pretreated electrodes, carbon fiber electrodes and self-assembled monolayers were made to elaborate reliable analytical tools for dopamine detection [39,40,41,42,43,44]. It should be stressed that boronic acids derivatives were also tested during the development of electrochemical sensors for the quantitative analysis of dopamine. Imprinted silica matrix-poly(aniline boronic acid) hybrid layers were formed on the surface of working electrodes for the selective and direct electrochemical detection of dopamine [45]. Dopamine-sensitive impedimetric responses were recorded for the electrodes functionalized with poly(3-thienylboronic acid) as well as phenyl-boronic acid, where [Fe(CN)_6_]^3−/4−^ reporter couple was added [46,47]. The selective recognition of dopamine by a ferrocene-based ditopic receptor bearing a boronic acid moiety and a benzo-18-crown-6-ether unit was recently reported, providing electrochemical sensing of the neurotransmitter [48]. Finally, another ditopic ligand based on hexahomotrioxacalix[3]arene with phenylboronic acid site was incorporated in polymer membranes of ion-selective electrodes [49].

The designs of various electrochemical sensors based on boronic acids derivatives were presented in our previous works. Lipophilic derivatives of phenylboronic acids were introduced as selective ionophores in potentiometric sensors dedicated to fluoride sensing [50] and to the analysis of amino acid mixtures [51], while thiolated boronic acids were exploited to form self-assembled monolayers on the surface of gold electrodes for the voltammetric detection of fructose [52]. Preliminary research on the application of organoboron receptors in polymeric membranes of ion-selective electrodes sensitive towards selected neurotransmitters was also undertaken [53]. In the present work, piperazine-based compounds with phenylboronic acid or benzoxaborole scaffolds were applied as ionophores in dopamine-sensitive ion-selective electrodes. The results were compared with those obtained for 4-octoxyphenylboronic acid. Additionally, the sensor selectivity was discussed in the context of DFT calculations.

## 2. Materials and Methods

### 2.1. Chemicals and Membrane Materials

**Figure 1:**
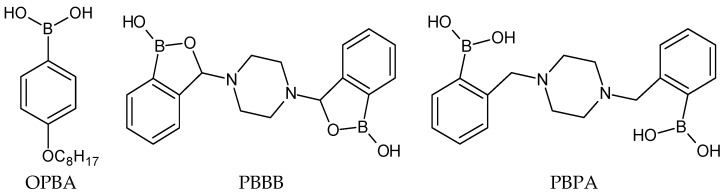
Structures of the studied organoboron receptors.

All sodium salts were of analytical grade and were purchased from Fluka. High-molecular-weight poly(vinylchloride) (PVC), bis(2-ethylhexyl) sebacate (DOS), o-nitrophenyl octyl ether (o-NPOE) and potassium tetrakis [3,5-bis(trifluoromethyl)phenyl] borate (KTFPB) were obtained from Fluka (Selectophore). Freshly distilled tetrahydrofuran from Fluka was used as a solvent for the membrane components. Dopamine, 2-phenylethylamine and acetylcholine were purchased from Sigma-Aldrich. Organoboron receptors 4-octyloxyphenylboronic acid (OPBA) [50,54], 3,3′-piperazine-bis(benzoxaborole) (PBBB) [33] and 3,3′-piperazine-bis(phenylboronic acid) PBPA [34] were synthesized according to the previously published procedures (Figure 1).

### 2.2. Ion-Selective Electrode Preparation and Electromotive Force (EMF) Measurements

The methods of membranes and electrodes preparation were the same as for classical liquid-membrane ion-selective electrodes [50]. The membranes contained: 2 wt % ionophore (organoboron receptor), 65 wt % plasticizer, 32–33 wt % PVC and 10 mol % (vs ionophore) KTFPB. The membrane components (200 mg in total) were dissolved in 2 mL of tetrahydrofuran. The solution was poured into a glass ring placed on a glass. After solvent evaporation, membrane discs of appropriate size were cut off and mounted in electrode bodies (type IS 561, Philips, Willi Möller AG, Zurich, Switzerland) for electromotive force (EMF) measurements. NaCl solution (0.1 M) was used as an internal filling. The electrodes were conditioned overnight in a dilute solution of internal electrolyte (0.01 M NaCl). For each membrane composition, three electrode specimens were prepared.

All potentiometric measurements were carried out with cells of the following type: Ag, AgCl; KCl 1 M/CH_3_COOLi 1 M/sample solution//membrane//internal filling solution; AgCl, Ag.

A potentiometric multiplexer (EMF 16 Interface, Lawson Labs Inc., Malvern, USA) was used for the EMF measurements. The calibration curves of the electrodes were examined by measuring the EMFs in buffered solution (pH 4.5), increasing the concentration of the neurotransmitter in steps of 0.5 log c (concentration range: 10^−6^–10^−2^ M). Potentiometric selectivity coefficients (log *K*_DOP, X_) of the polymeric membranes were determined by the separate solution method (SSM) using 0.01 M solutions. The activities of anions in aqueous solutions were calculated according to the Debye–Hückel approximation.

### 2.3. Computational Methods

The single-molecule geometry optimizations were performed according to the DFT method using M06-2X Minnesota Functional [55,56] and 6-311+G(d,p) [57] basis-set implemented in Gaussian09 program [58]. The minima were confirmed by vibrational frequency calculations within the harmonic approximation (no imaginary frequencies). During the calculations no symmetry constraints were applied. The receptor–analyte complexation energies were calculated as the difference between energies of complex and separate components. In all cases single-molecule binding scheme was assumed. The obtained values were corrected for the basis-set superposition error using the counterpoise procedure. A Polarizable Continuum Model (PCM) [59,60,61,62] with a dielectric constant of 3.9 was used to imitate the plasticizer environment (DOS).

## 3. Results and Discussion

### 3.1. Sensitivity and Selectivity of Potentiometric Sensors

In this work, three organoboron compounds of various structures were applied as dopamine receptors of potentiometric sensors. 4-Octyloxyphenylboronic acid (OPBA) was proposed as a model ionophore, used previously in ion-selective electrodes sensitive towards fluoride anions and in sensor arrays of miniaturized electrodes for quantitative amino acids analysis [50,51]. Moreover, piperazine-based symmetrical organoboron species bearing two phenylboronic acid or two benzoxaborole groups (PBPA and PBBB) were selected due to their improved selectivity and significantly different Lewis acidity of their boronic centers (*pK*_a_ = 8.7 and 7.3, respectively) [14,63].

**Figure 2:**
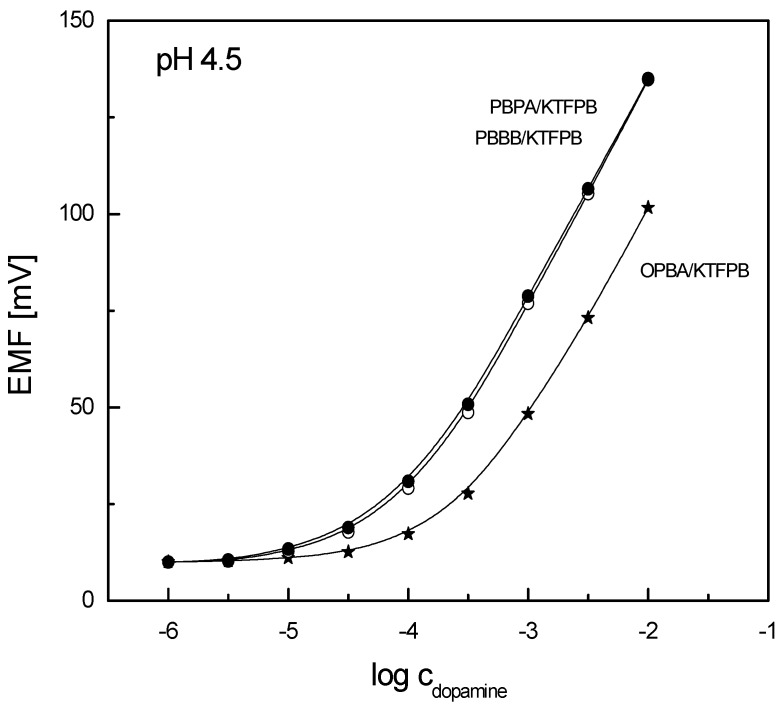
Potentiometric responses of ion-selective electrodes formulated with organoboron receptors (PVC/DOS, 10 mol% KTFPB) towards dopamine.

The performances of the potentiometric sensors were evaluated in buffered solutions at pH 4.5, since dopamine remains in its protonated form in such conditions (*pK*_a_ = 8.93). The ion-selective electrodes formulated with studied organoboron receptors in the PVC/DOS membranes containing KTFPB anionic additives were found to respond potentiometrically to dopamine (Figure 2). Similar, high sensitivity was noticed for the sensors based on bifunctional PBPA and PBBB ionophores; their response slopes were close to the theoretical value (see Table 1). On the other hand, a limited linear range of the calibration curves (with comparable sensitivity) was recorded in the case of electrodes with the model OPBA receptor. This result indicated that the addition of the second boronic acid or benzoxaborole moiety to the receptor structure may alter its interaction not only with saccharides, as postulated in the literature [64]. It should be stressed that the presence of KTFPB in the polymer membranes significantly increased the response sensitivity as well as improved the detection limit of the sensors (the responses of electrodes without anionic additives were measured in quite a narrow linear range—results not shown, see Table 1).

**Figure 3:**
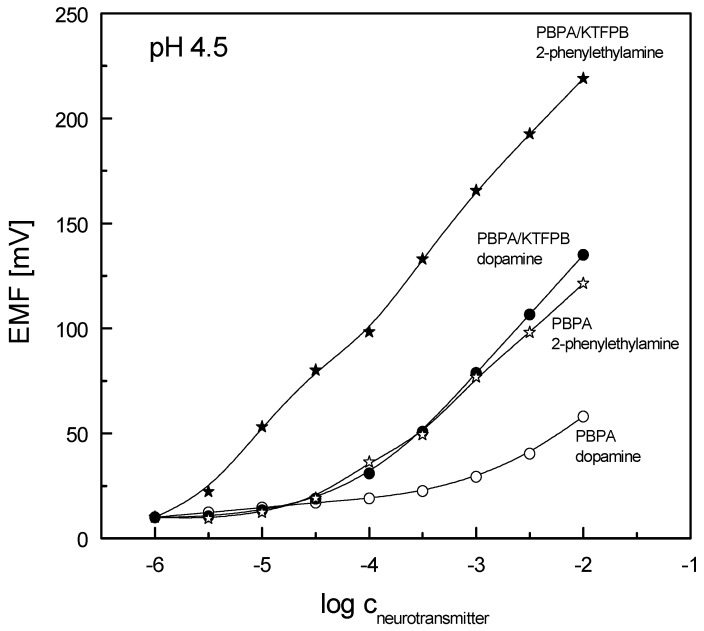
Potentiometric responses of ion-selective electrodes formulated with PBPA receptor (PVC/DOS, with and without KTFPB) towards dopamine and 2-phenylethylamine.

Next, the potentiometric responses towards dopamine were compared with those registered for 2-phenylethylamine, a neurotransmitter of similar structure, that in absence of the catechol moiety may interact with organoboron-based species only through the dative B–N bond. Exemplary characteristics of electrodes based on PBPA (with and without KTFBP in membranes) are presented in Figure 3 (very similar performances were received for the electrodes containing PBBB receptor in membranes). The values of sensitivity, limit of detection and linear range of the potentiometric responses of ion-selective electrodes towards dopamine and 2-phenylethylamine are compared in Table 1. A distinctly wider linear range of the calibration curves was observed towards 2-phenylethylamine, with near-Nernstian sensitivity. The differences in values of membrane potential, measured in solutions of both neurotransmitters, suggested electrode selectivity towards 2-phenylethylamine over dopamine and were consistent with the results reported for PVC membranes containing a ditopic hexahomotrioxacalix[3]arene derivative with a phenylboronic acid substituent [49]. As previously stated, the introduction of KTFBP in the membranes promoted higher sensitivity and lowered the detection limit of the electrodes.

Finally, the potentiometric selectivity coefficients of the polymer membranes doped with the studied organoboron receptors towards dopamine over selected neurotransmitters (2-phenylethylamine, acetylcholine) and inorganic cations were determined (Table 2). Regardless of the structure of the ionophore, the ion-selective electrodes exhibited selectivity for dopamine over inorganic cations, with strong interferences from 2-phenylethylamine (comparable sensor selectivity was reported in Ref. [49]). Similar selectivity patterns (as well as the sensitivity described above) of the membranes formulated with bifunctional compounds pointed out that the difference in the Lewis acidity of such receptors did not influence the host–guest interactions in the membrane environment. Moreover, according to our expectations, the electrodes without anionic additives in the membranes exhibited limited dopamine preference over inorganic cations, which was improved by the presence of KTFPB (see the comparison of log *K*_DOP, X_ values calculated for membranes based on PBPA in Table 2). Comparable selectivity patterns were obtained for membranes plasticized with highly polar o-NPOE.

To conclude, it is difficult to elucidate the origin of 2-phenylethylamine/dopamine preferences of the developed sensors, since the calculated potentiometric selectivity reflects both the differences in the affinity of the receptors to various neurotransmitters and the lipophilicity of the sensed molecules.

### 3.2. Receptor–Analyte Interaction—A Computational Approach

**Figure 4:**
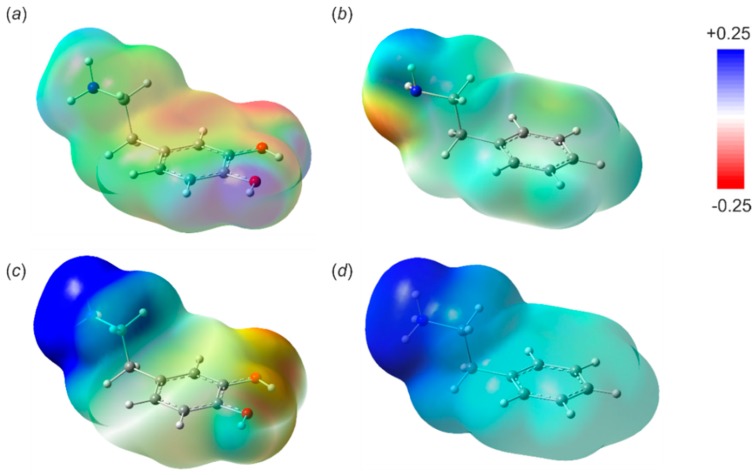
Semi-transparent representation of electrostatic potential (eÅ^−1^) mapped onto the electron density surface (ρ = 0.003 eÅ^−3^) generated for: (**a**) dopamine, (**b**) phenylethylamine and their corresponding cationic forms (**c**,**d**).

In order to investigate the origin of observed sensitivity and the selectivity of organoboron-based ion-selective electrodes towards 2-phenylethylamine and dopamine, a series of single-molecule theoretical calculations on the M06-2X [55,56]/6-311+G(d,p) [57] level of theory was undertaken. Calculations were performed using a Polarizable Continuum Model [59,60,61,62] with a dielectric constant of 3.9 to mimic the environment of the plasticizer (DOS). To simplify the considerations, we assumed that the electrode response strongly depends on analyte diffusion kinetic through the PVC membrane and the receptor–analyte association constants. Regarding the transportation process, it was expected that dopamine would be significantly less lipophilic than 2-phenylethylamine due to the presence of two hydroxyl groups. Indeed, the electrostatic potential mapped onto the electron density surface showed strong charge distribution variations in dopamine molecules, while in the case of 2-phenylethylamine the electrostatic potential was more uniform, with some charge variations observed around the amine group (see Figure 4). Essentially, a similar conclusion can be derived from the comparison of corresponding ammonium forms. This also stays in agreement with the experimental octanol-water partition coefficients (log), equal to −0.98 for dopamine and 1.41 for 2-phenyl-ethylamine.

In the next step, the receptor (OPBA, PBBB, PBPA)–analyte (dopamine, 2-phenylethylamine) complexation energies were calculated and compared (ΔE). The relevant values are given in Table 3. 2-Phenylethylamine binds to the diboronic acid/dibenzoxaborole derivatives through the dative B–N bond (i.e., between the boronic center and the NH_3_^+^ group of dopamine), leading to the rehybridization of the boron coordination sphere to tetrahedral form. Dopamine possesses two available binding sites: an amine nitrogen atom and two aromatic hydroxyl groups. The latter groups form catechol ester with boronic acid in accordance with the well-described boronic acid–diol complexation mechanism under aqueous acidic conditions.

According to our calculations, the PBPA molecules exhibited the strongest analyte binding capabilities. Regardless of the binding scheme, the dopamine binding selectivity followed the experimentally observed order: PBPA > PBBB > OPBA. Furthermore, the B–N complexation was energetically favored over the catechol binding in the case of PBPA and PBBB, whereas the formation of catechol ester was more preferred for the OPBA derivative. The observed relation is a consequence of the boron Lewis acidity and the rigidity of the boronic group. OPBA, being the weakest Lewis acid in the studied series (with *p*K_a_ of about 9.5 [65]), weakly interacts with amines. In turn, the PBPA molecule represents the Wulf-type receptor with two relatively strong intramolecular OH–N hydrogen bonds, found both in the theoretically optimized molecule and crystal structures of this compound [34]. In the case of PBBB, the rigidity of the oxaborle ring imposes a higher boron Lewis acidity in accordance with the well-recognized structural effect of the five-membered oxaborole ring [66].

Finally, the calculated receptor–analyte complex formation energies proved that the complexation of 2-phenylethylamine is slightly more advantageous, compared to that of dopamine. This stays in agreement with the experimental results received for PBPA. However, since the difference in complex formation energies does not exceed 3 kJ·mol^−1^, the measured electrode selectivity towards 2-phenylethylamine over dopamine resulted from lipophilicity differences.

## 4. Conclusions

Ion-selective electrodes based on receptors bearing two phenylboronic acid or benzoxaborole groups were tested for dopamine sensing. The sensitivity and selectivity of the sensors towards dopamine was observed, but strong interferences from 2-phenylethylamine were unexpected. Moreover, the introduction of bifunctional organoboron ionophores to the polymer membranes appreciably improved the detection limit of the constructed electrodes. This result suggested that two symmetrical organoboron moieties in the receptor structure could be beneficial not only for the modification of the sensor selectivity towards saccharides.

The results were consistent with those reported for ion-selective electrodes based on the ditopic hexahomotrioxacalix[3]arene derivative-bearing boronic acid substituent [49]. In both cases, the favorable binding affinity of single boronic acid receptors to dopamine did not ensure the advantageous dopamine/2-phenyl-ethylamine sensor selectivity. However, the use of ionophores possessing double organoboron moieties provided to a certain extent the reduction of interferences from 2-phenylethylamine as compared with the calix[3]arene-based derivative.

Next, single-molecule theoretical calculations were performed in order to elucidate the origin of 2-phenylethylamine/dopamine selectivity. The results revealed that dopamine complexation through the dative B–N bond is energetically more favored than the formation of catechol ester in the case of bifunctional receptors (PBPA and PBBB), whereas the catechol binding is more preferred for OPBA. Therefore, similar interactions can be considered when discussing the selectivity of membranes, containing PBPA or PBBB, toward dopamine and 2-phenylethylamine. Despite the fact that the calculations showed higher affinities of bifunctional receptors to 2-phenylethylamine, the determined potentiometric selectivity can be rationalized in terms of the different lipophilic characters of guest molecules.

To conclude, the results obtained clearly demonstrated that, taking into account the lipophilicity of the neurotransmitters, the synthesis of an organoboron derivative strongly favoring dopamine binding is required to develop potentiometric sensors that can exhibit the desired discrimination between dopamine and 2-phenylethylamine.

## Figures and Tables

**Table 1:** Values of sensitivity, limit of detection and linear range of the potentiometric responses of ion-selective electrodes formulated with the studied organoboron receptors (with and without KTFPB in membranes) towards: (**a**) dopamine and (**b**) 2-phenylethylamine.

	Sensitivity (mV/dec)	Linear Range (M)	Detection Limit (M)
OPBA ^(a)^	-	-	-
OPBA/KTFPB ^(a)^	53.5	10^−3^ ÷ 10^−2^	2 × 10^−4^
PBBB ^(a)^	9.5	10^−3^ ÷ 10^−2^	3 × 10^−4^
PBBB/KTFPB ^(a)^	56.5	3 × 10^−4^ ÷ 10^−2^	8 × 10^−5^
PBPA ^(a)^	27.5	10^−3^ ÷ 10^−2^	6 × 10^−4^
PBPA/KTFPB ^(a)^	56.0	3 × 10^−4^ ÷ 10^−2^	8 × 10^−5^
PBPA ^(b)^	47.0	3 × 10^−4^ ÷ 10^−2^	8 × 10^−5^
PBPA/KTFPB ^(b)^	56.5	10^−5^ ÷ 10^−2^	3 × 10^−6^

**Table 2:** Values of selectivity coefficients (log *K*_DOP, X_) of ion-selective electrodes formulated with studied organoboron receptors with 10 mol % KTFPB and without KTFPB in membranes (mean values calculated for three electrode specimens).

log *K*_DOP, X_	OPBA/KTFPB	PBBB/KTFPB	PBPA/KTFPB	PBPA
2-Phenylethylamine	-	1.70	1.70	1.60
Acetylcholine	0.45	0.40	0.45	0.20
Dopamine	0.00	0.00	0.00	0.00
Na^+^	−1.35	−1.40	−1.35	−1.10
K^+^	−1.00	−0.95	−0.90	−0.70
NH_4_^+^	−2.45	−2.40	−2.45	−1.70
Ca^2+^	−2.50	−2.65	−2.70	−1.90

**Table 3:** Receptor–analyte complex formation energies (ΔE). In the case of dopamine, two different complexation modes (through B–N interaction and catechol ester formation) are presented.

Δ*E*/kJ mol^−1^	OPBA	PBBB	PBPA
PhEtNH_2_ (B–N)	−6.7	−16.1	−21.2
Dopamine (B–N)	−6.6	−15.0	−18.3
Dopamine (catechol)	−12.9	−8.1	−8.3
